# Comparative Anti-inflammatory Activity of Silver and Zinc Oxide Nanoparticles Synthesized Using Ocimum tenuiflorum and Ocimum gratissimum Herbal Formulations

**DOI:** 10.7759/cureus.52995

**Published:** 2024-01-26

**Authors:** Remmiya Mary Varghese, Aravind Kumar S, Rajeshkumar Shanmugam

**Affiliations:** 1 Orthodontics and Dentofacial Orthopedics, Saveetha Dental College and Hospitals, Saveetha Institute of Medical and Technical Sciences, Saveetha University, Chennai, IND; 2 Nanobiomedicine Lab, Centre for Global Health Research, Saveetha Medical College and Hospital, Saveetha Institute of Medical and Technical Sciences, Chennai, IND; 3 Pharmacology, Saveetha Dental College and Hospitals, Saveetha Institute of Medical and Technical Sciences, Chennai, IND

**Keywords:** eco-friendly, anti-inflammatory, antimicrobial agent, green synthesis, zinc oxide nanoparticles

## Abstract

Background

The aim of this study was to evaluate and compare the anti-inflammatory properties of silver nanoparticles (AgNPs) and zinc oxide nanoparticles (ZnONPs) that were synthesized utilizing African tulsi and black tulsi herbal formulations. The anti-inflammatory activity was assessed by the utilization of bovine serum albumin (BSA) denaturation and egg albumin denaturation tests. In addition, a membrane stabilization experiment was performed to evaluate their efficacy as anti-inflammatory drugs*.*

Methods

This study was conducted at Saveetha Dental College and Hospitals, Saveetha Institute of Medical and Technical Sciences (SIMATS), Saveetha University, Chennai, India. AgNPs and ZnONPs were synthesized using *Ocimum tenuiflorum* (African tulsi) and *Ocimum gratissimum* (black tulsi) extracts. The BSA denaturation assay involved mixing serum albumin with different nanoparticle concentrations (10-50 µg/mL) and measuring absorbance at 660 nm. The egg albumin denaturation assay followed a similar procedure. The membrane stabilization assay utilized red blood cells and spectrophotometric measurements at 540 nm.

Results

In the BSA denaturation assay, AgNPs and ZnONPs showed concentration-dependent inhibition of protein denaturation. While these nanoparticles exhibited anti-inflammatory potential, diclofenac sodium consistently displayed slightly stronger inhibition. In the egg albumin denaturation assay, AgNPs and ZnONPs inhibited protein denaturation at various concentrations. Their anti-inflammatory effects were comparable to the standard drug, diclofenac sodium. In the membrane stabilization assay, both nanoparticle types demonstrated concentration-dependent membrane stabilization effects. Diclofenac sodium exhibited slightly stronger membrane stabilization.

Conclusions

AgNPs and ZnONPs synthesized using *Ocimum tenuiflorum* and *Ocimum gratissimum* (African tulsi and black tulsi) possess anti-inflammatory potential, as demonstrated by their inhibition of protein denaturation and membrane stabilization. While these nanoparticles show promise as anti-inflammatory agents, further research is needed to explore their clinical applications and safety profiles.

## Introduction

Silver nanoparticles (AgNPs) have garnered significant attention due to their diverse range of applications across various fields. In the biotechnology and medical sectors, they have become a focal point of research for their potential therapeutic properties, which encompass anticancer, anti-inflammatory, antibacterial, antiviral, and anti-angiogenic effects [1]. AgNPs have also made substantial contributions to diagnostic tools, medicinal equipment, drug delivery systems, imaging agents, and coatings for biomedical devices [2]. Moreover, their versatility extends to nanomedicine, drug delivery, electronics, the energy sector, and environmental protection [3].

One notable aspect of AgNPs is their biogenic synthesis, often referred to as bionanoparticles, which presents a compelling option. These nanoparticles are cost-effective, non-toxic, and sustainable, making them highly suitable for a wide array of biomedical applications [4]. In the field of dentistry, AgNPs find utility in disinfection, the prevention of oral infections, the elimination of plaque and calculus, and the treatment of bacterial and fungal oral issues [5]. Overall, AgNPs exhibit diverse applications in biotechnology, medicine, diagnostics, dentistry, and other areas, making them a promising subject for continued research and development.

On the other hand, zinc oxide nanoparticles (ZnONPs) have also emerged as a versatile player in both biomedicine and dentistry. In dentistry, ZnONPs play a pivotal role in enhancing the antibacterial properties of restorative materials, acting as anti-sensitivity agents in toothpaste formulations, and augmenting the anti-fungal effects of denture bases. They are also valuable as coatings for dental implants and contribute to the remineralization of cervical dentinal lesions [6]. In the realm of biomedicine, ZnONPs serve a multitude of purposes, including their application in anticancer therapies, antibacterial treatments, and drug/gene delivery systems [7,8]. These nanoparticles exhibit the remarkable ability to generate reactive oxygen species (ROS) and induce programmed cell death, rendering them effective against pathogens responsible for dental decay. Importantly, ZnONPs can be synthesized through environmentally friendly biological means, reducing their toxicity to human health and enhancing their appeal [9].

Shifting the focus to herbal extracts, *Ocimum tenuiflorum*, or black tulsi, is a revered medicinal plant with a wide range of health benefits. Rooted in traditional Ayurvedic medicine, it is considered an adaptogen and has been used to treat various ailments. The plant is rich in active chemical constituents, including terpenoids, phenols, flavonoids, and essential oils, which contribute to its therapeutic properties. These compounds have demonstrated potential in addressing conditions, such as diabetes, inflammation, microbial infections, and cardiovascular disorders [10,11]. Furthermore, tulsi's essential oil exhibits antioxidant properties, making it a valuable ingredient for use in the cosmetics and food industries. In addition, the plant has been identified as a potential source of methyl eugenol, which has applications in fruit fly management in Africa [12]. Therefore, tulsi stands as a versatile plant with diverse pharmacological actions and promising applications across various fields [13].

Similarly, *Ocimum grattissimum*, also known as African tulsi, is a medicinal plant renowned for its therapeutic attributes. It has found applications in various branches of medicine, particularly oral medicine, due to its analgesic, anti-inflammatory, antibacterial, and anti-ulcerative properties [14]. Tulsi may be beneficial in the prevention of a range of oral health issues, such as tooth decay, pain, gingivitis, halitosis, and periodontitis [15]. The plant's therapeutic prowess can be attributed to its diverse array of active chemical constituents, including terpenoids, phenols, flavonoids, and essential oils [16]. In addition to its oral health benefits, tulsi exhibits pharmacological effects that alleviate stress, protect organs and tissues, and promote a sense of well-being. Furthermore, its versatile antibacterial properties make it valuable in applications, such as water purification, wound healing, and food preservation [17].

In this present study, we synthesized African tulsi and black tulsi herbal formulation-mediated AgNPs and ZnONPs and tested for their anti-inflammatory activity using assays like the bovine serum albumin (BSA) denaturation assay, egg albumin denaturation assay, and membrane sterilization assay.

## Materials and methods

This study was conducted at Saveetha Dental College and Hospitals, Saveetha Institute of Medical and Technical Sciences (SIMATS), Saveetha University, Chennai, India.

Preparation of herbal formulation

One gram of *Ocimum tenuiflorum* and 1 g of *Ocimum gratissimum* were accurately added to 100 mL distilled water. The mixture was then subjected to heating using a heating mantle at a temperature of 60°C for 15-20 minutes. Subsequently, the boiled mixture underwent a gradual filtration process utilizing filter paper. The resulting filtrate, containing the extract, was then stored for subsequent nanoparticle synthesis.

Green synthesis of ZnONPs and AgNPs

The synthesis of ZnONPs using a green approach involving African basil and black tulsi (*Ocimum tenuiflorum* and *Ocimum grattissimum*) extracts in the presence of a zinc nitrate solution (30 mM in 50 mL distilled water) was undertaken in this study. This environmentally friendly method harnesses the bioactive compounds present in these herbal extracts for the reduction and stabilization of ZnONPs. The procedure began with the preparation of a zinc nitrate solution, providing a controlled source of zinc ions. Subsequently, 50 mL of African basil and black tulsi extract, known for their rich phytochemical content, was combined with the zinc nitrate solution. Similarly, for the green synthesis of AgNPs, a 1 mM silver nitrate solution was prepared by dissolving silver nitrate in 80 mL distilled water. To this solution, 20 mL of a filtered herbal formulation extract was added. The resulting mixtures were subjected to a centrifugation process at 8000 rpm for 10 minutes.

The centrifugation step played a pivotal role in both ZnONPs and AgNPs' synthesis process. It facilitated the separation of synthesized nanoparticles from any unreacted precursors or extract residues. The pellet collected after centrifugation represents the desired ZnONPs and AgNPs, which were then subjected to further characterization and evaluation.

Anti-inflammatory activity

The in vitro anti-inflammatory activity was performed by assays, such as the BSA denaturation assay, egg albumin denaturation assay, and membrane stabilization assay, as reported in a previous work by Harris et al. [18].

BSA denaturation assay

A solution containing 0.45 mL of BSA was combined with 0.05 mL of AgNPs and ZnONPs and generated using a green method, with concentrations ranging from 10 to 50 µg/mL. The pH was modified to a value of 6.3. Subsequently, it was stored at ambient temperature for a duration of 10 minutes and then subjected to incubation in a water bath set at 55°C for a period of 30 minutes. The standard group was administered diclofenac sodium, while the control group was administered dimethyl sulphoxide. Subsequently, the samples were quantified using spectrophotometry at a wavelength of 660 nm.

The percentage of protein denaturation was determined using the following equation:

% inhibition = (Absorbance of control - Absorbance of sample × 100) / Absorbance of control

Egg albumin denaturation assay

In order to conduct the egg albumin denaturation test, 0.2 mL of freshly obtained egg albumin was combined with 2.8 mL of phosphate buffer. Various amounts (ranging from 10 to 50 µg/mL) of African basil and black tulsi were used to synthesize AgNPs and ZnONPs, which were then added to the reaction mixture. The pH was modified to a value of 6.3. Subsequently, the sample was allowed to equilibrate at ambient temperature for a duration of 10 minutes and then subjected to incubation in a water bath set at a temperature of 55°C for a period of 30 minutes. The standard group was treated with diclofenac sodium, whereas the control group was treated with dimethyl sulphoxide. Subsequently, the samples were quantified using spectrophotometry at a wavelength of 660 nm.

The percentage of protein denaturation was determined using the following equation:

% inhibition = (absorbance of control - absorbance of sample × 100) / absorbance of control

Membrane stabilization assay

The in vitro membrane stabilization assay is a commonly employed method for assessing the membrane stabilizing characteristics of both natural and synthesized substances. This assay quantifies the capacity of a chemical to enhance the stability of the cell membrane by inhibiting its disruption and subsequent leakage of intracellular contents. The ingredients consist of human red blood cells (RBCs), phosphate-buffered saline (PBS), tris-HCl buffer (50 mM, pH 7.4), various quantities of green-produced AgNPs and ZnONPs (10-50 µg/mL), a centrifuge tube, and a UV-Vis spectrophotometer.

Preparation of RBC suspension

To prepare a suspension of RBCs, obtain a sample of recently drawn human blood and place it in a sterile tube that contains an anticoagulant. Apply centrifugal force to the blood at a speed of 1000 times the acceleration due to gravity for a duration of 10 minutes at the ambient temperature in order to segregate the RBCs from the rest of the blood constituents. Discard the liquid portion and cleanse the RBCs by rinsing them three times with PBS. Reconstitute the RBCs in tris-HCl buffer to get a 10% (v/v) RBC suspension.

Assay procedure

Transfer 1 milliliter of the RBC suspension into each centrifuge tube using a pipette. Subsequently, varying amounts of African basil and black tulsi were introduced to individual tubes, along with AgNPs and ZnONPs. Stir the contents carefully and place the tubes in a controlled environment at a temperature of 37°C for a duration of 30 minutes. To separate the RBCs, subject the tubes to centrifugal force of 1000 g for 10 minutes at room temperature. Utilize a UV-Vis spectrophotometer to quantify the absorbance of the supernatant at a wavelength of 540 nm.

Determine the % inhibition of hemolysis by applying the given formula:

[(OD control - OD sample) / OD control] x 100, where *OD control* represents the absorbance of the RBC suspension without the test chemical(s) and *OD sample* represents the absorbance of the RBC suspension with the test compound.

## Results

Anti-inflammatory activity of AgNPs

**Figure 1:**
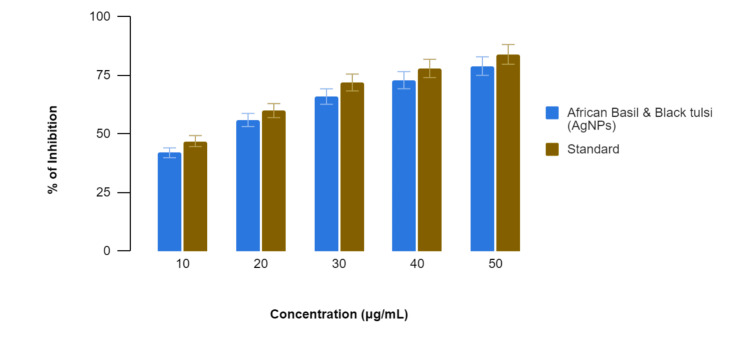
Anti-inflammatory activity of green-mediated AgNPs using the bovine serum albumin denaturation assay AgNPs: silver nanoparticles

In Figure 1, the BSA denaturation assay, African basil- and black tulsi-mediated AgNPs exhibited inhibitory effects on BSA denaturation at various concentrations. By contrast, diclofenac sodium, a common anti-inflammatory drug, consistently showed a slightly stronger ability to stop BSA denaturation at all concentration levels. Specifically, at 10 µg/mL, the African basil- and black tulsi-mediated AgNPs showed 42% inhibition compared to 47% by diclofenac sodium. At 20 µg/mL, the inhibition percentages were 56% for the African basil- and black tulsi-mediated AgNPs and 60% for diclofenac sodium; at 30 µg/mL, they were 66% and 72%, respectively; at 40 µg/mL, they were 73% and 78%, respectively; and at 50 µg/mL, they were 79% and 84%, respectively. While African basil and black tulsi (AgNPs) demonstrated potential anti-inflammatory activity at all concentrations, diclofenac sodium consistently displayed a slightly stronger inhibitory effect on BSA denaturation across the concentration range.

**Figure 2:**
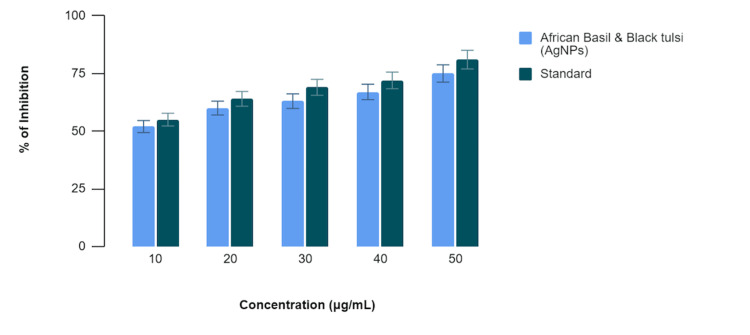
Anti-inflammatory activity of green-mediated AgNPs using the egg albumin denaturation assay AgNPs: silver nanoparticles

In Figure 2, the egg albumin denaturation assay, the African basil- and black tulsi-mediated AgNPs exhibited varying degrees of inhibition of egg albumin denaturation at different concentrations. Specifically, at 10 µg/mL, the African basil- and black tulsi-mediated AgNPs displayed 52% inhibition compared to 55% by the standard anti-inflammatory drug (diclofenac sodium). At 20 µg/mL, the percentages were 60% for the African basil- and black tulsi-mediated AgNPs and 64% for diclofenac sodium; at 30 µg/mL, they were 63% and 69%, respectively; at 40 µg/mL, they were 67% and 72%, respectively; and at 50 µg/mL, they were 75% and 81%, respectively. While the African basil- and black tulsi-mediated AgNPs demonstrated anti-inflammatory potential at all concentrations, the standard consistently exhibited slightly stronger inhibition of egg albumin denaturation across the concentration range.

**Figure 3:**
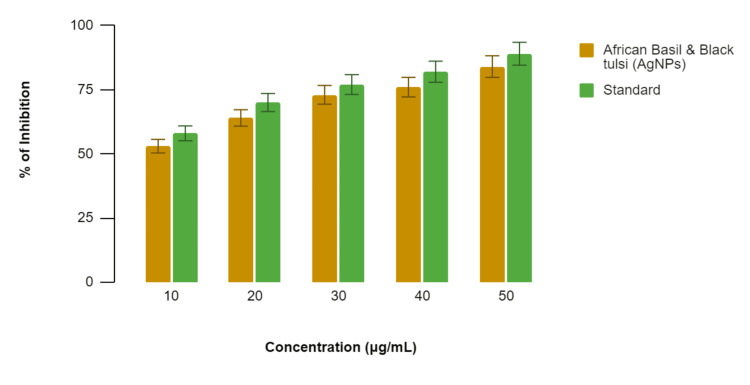
Anti-inflammatory activity of green-mediated AgNPs using the membrane stabilization assay AgNPs: silver nanoparticles

In Figure 3, the membrane stabilization assay for the African basil- and black tulsi-mediated AgNPs demonstrated varying degrees of membrane stabilization at different concentrations, while the standard anti-inflammatory drug (diclofenac sodium) also displayed membrane stabilization. Specifically, at 10 µg/mL, the African basil- and black tulsi-mediated AgNPs exhibited 53% membrane stabilization compared to 58% by diclofenac sodium. At 20 µg/mL, the percentages were 64% for the African basil- and black tulsi-mediated AgNPs and 70% for diclofenac sodium; at 30 µg/mL, they were 73% and 77%, respectively; at 40 µg/mL, they were 76% and 82%, respectively; and at 50 µg/mL, they were 84% and 89%, respectively. While the African basil- and black tulsi-mediated AgNPs demonstrated anti-inflammatory potential with membrane stabilization at all concentrations, the standard consistently exhibited a slightly stronger membrane stabilization effect across the concentration range.

Anti-inflammatory activity of ZnONPs

**Figure 4:**
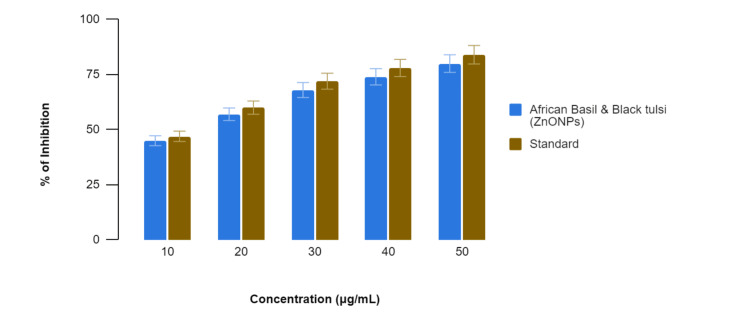
Anti-inflammatory activity of the Ocimum tenuiflorum- and Ocimum gratissimum-mediated ZnONPs using the bovine serum albumin denaturation assay ZnONPs: zinc oxide nanoparticles

In Figure 4, in the BSA denaturation assay, the African basil- and black tulsi-mediated ZnONPs displayed inhibitory effects on BSA denaturation at various concentrations. Comparatively, the standard anti-inflammatory drug exhibited similar inhibitory effects on BSA denaturation. Specifically, at 10 µg/mL, the African basil- and black tulsi-mediated ZnONPs showed 45% inhibition, while the standard anti-inflammatory drug displayed 47% inhibition. At 20 µg/mL, the percentages were 57% for the African basil and black tulsi-mediated ZnONPs and 60% for the standard anti-inflammatory drug; at 30 µg/mL, they were 68% and 72%, respectively; at 40 µg/mL, they were 74% and 78%, respectively; and at 50 µg/mL, they were 80% and 84%, respectively. These results suggest that the African basil- and black tulsi-mediated ZnONPs exhibit anti-inflammatory potential by inhibiting BSA denaturation, with inhibitory effects comparable to those of the standard across all concentrations tested.

**Figure 5:**
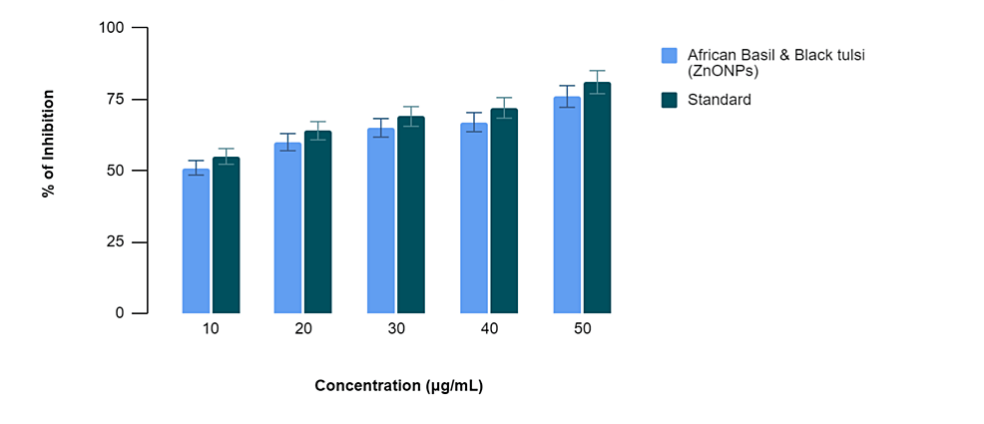
Anti-inflammatory activity of the Ocimum tenuiflorum- and Ocimum gratissimum-mediated ZnONPs using the egg albumin denaturation assay ZnONPs: zinc oxide nanoparticles

In Figure 5, in the egg albumin denaturation assay, the African basil- and black tulsi-mediated ZnONPs exhibited varying degrees of inhibition of egg albumin denaturation at different concentrations, while the standard anti-inflammatory drug, diclofenac sodium, also displayed inhibition. Specifically, at 10 µg/mL, the African basil- and black tulsi-mediated ZnONPs demonstrated 51% inhibition compared to 55% by diclofenac sodium. At 20 µg/mL, the percentages were 60% for the African basil and black tulsi-mediated ZnONPs and 64% for diclofenac sodium; at 30 µg/mL, they were 65% and 69%, respectively; at 40 µg/mL, they were 67% and 72%, respectively; and at 50 µg/mL, they were 76% and 81%, respectively. These results indicate that the African basil- and black tulsi-mediated ZnONPs possess anti-inflammatory potential by inhibiting egg albumin denaturation, with inhibitory effects comparable to those of the standard, diclofenac sodium, across all concentrations tested, suggesting their potential as anti-inflammatory agents.

**Figure 6:**
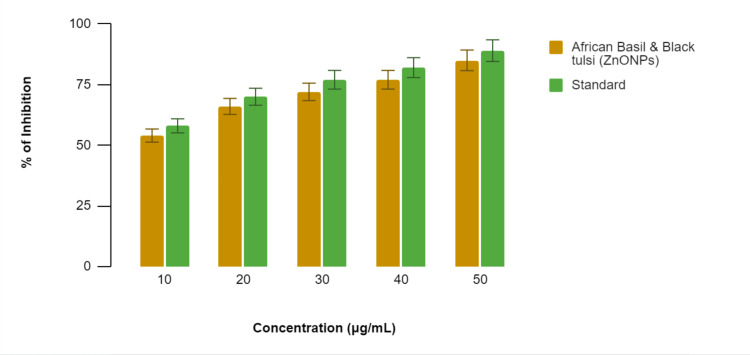
Anti-inflammatory activity of the Ocimum tenuiflorum- and Ocimum gratissimum-mediated ZnONPs using the membrane stabilization assay ZnONPs: zinc oxide nanoparticles

In Figure 6, in the membrane stabilization assay, the African basil- and black tulsi-mediated ZnONPs displayed varying degrees of membrane stabilization at different concentrations, while the standard anti-inflammatory drug, diclofenac sodium, also exhibited membrane stabilization effects. Specifically, at 10 µg/mL, the African basil- and black tulsi-mediated ZnONPs demonstrated 54% membrane stabilization compared to 58% by diclofenac sodium. At 20 µg/mL, the percentages were 66% for the African basil- and black tulsi-mediated ZnONPs and 70% for diclofenac sodium; at 30 µg/mL, they were 72% and 77%, respectively; at 40 µg/mL, they were 77% and 82%, respectively; and at 50 µg/mL, they were 85% and 89%, respectively. These results indicate that the African basil- and black tulsi-mediated ZnONPs possess anti-inflammatory potential by stabilizing cell membranes, with membrane stabilization effects comparable to those of the standard, diclofenac sodium, across all concentrations tested, suggesting their potential as anti-inflammatory agents.

## Discussion

In recent years, nanomaterials have gained substantial attention for their potential as anti-inflammatory agents. This discussion focuses on the comparative analysis of AgNPs and ZnONPs synthesized from African basil (*Ocimum gratissimum*) and black tulsi (*Ocimum gratissimum*) extracts, with a specific emphasis on their anti-inflammatory properties. The insights provided here aim to elucidate the potential therapeutic value of these nanoparticles in mitigating inflammation and their suitability for various applications. In the BSA denaturation assay, both AgNPs and ZnONPs showed anti-inflammatory activity, which showed that they might be able to stop proteins from becoming denaturated. It is worth noting that AgNPs consistently displayed a slightly higher degree of inhibition, particularly at lower concentrations (10 and 20 µg/mL). This suggests that AgNPs may have a modest advantage in preventing protein denaturation, pointing toward their potential efficacy in reducing inflammation. Similarly, in the egg albumin denaturation assay, AgNPs consistently outperformed ZnONPs, particularly at lower concentrations. At concentrations of 10 and 20 µg/mL, AgNPs exhibited superior inhibition of egg albumin denaturation when compared to ZnONPs. These findings reinforce the notion that AgNPs might possess a slight edge in anti-inflammatory efficacy, especially when employed at lower concentrations. The membrane stabilization assay yielded promising results for both AgNPs and ZnONPs synthesized from African basil and black tulsi extracts. Both types of nanoparticles effectively stabilized cell membranes, with no significant difference observed between them. This indicates that both AgNPs and ZnONPs can reliably stabilize cell membranes, suggesting their equal potential in this aspect of anti-inflammatory activity.

Expanding on the discussion, it is important to note that AgNPs have been extensively studied for their antimicrobial properties, but recent research has also uncovered their anti-inflammatory potential. Nguyen et al. [19] conducted a study demonstrating the anti-inflammatory activity of AgNPs synthesized with *Azadirachta indica* aqueous kernel extract. The research found that these AgNPs exhibited anti-inflammatory activity comparable to standard drugs when administered at a concentration of 100 μg mL^−1^. This suggests the potential utility of AgNPs in managing inflammation. However, Shehensha and Jyothi [20] reported that while AgNPs have anti-inflammatory benefits, they can also have pro-inflammatory effects when reactive oxygen species (ROS) are involved. These contrasting effects underscore the need for a comprehensive understanding of AgNP mechanisms and contexts of use in anti-inflammatory applications. In addition, Ansari et al. [21] investigated AgNPs synthesized using cinnamon oil and observed potent anti-inflammatory activity compatible with that of diclofenac sodium, a standard anti-inflammatory drug. These findings further support the potential of AgNPs as effective anti-inflammatory agents. On the other hand, ZnONPs have shown promise in fighting inflammation. Studies have shown that they can be made in a green way using plant extracts, such as *Senecio chrysanthemoides*, *Euphorbia retusa*, and *Kalanchoe pinnata* [22]. These biosynthesized ZnONPs have been shown to reduce inflammation through mechanisms that involve activating caspase-8 and stopping the production of cytokines that cause inflammation. ZnONPs have also been found to be compatible with anti-inflammatory drugs, such as diclofenac sodium, and have shown potential as drug delivery vectors. ZnONPs synthesized through green methods present themselves as effective and biocompatible anti-inflammatory agents [23,24].

AgNPs made from African basil and black tulsi extracts seem to be a little better at stopping protein denaturation at lower concentrations than ZnONPs, according to the data. However, these differences in anti-inflammatory activity are relatively small and may not have substantial clinical implications. The choice between AgNPs and ZnONPs for anti-inflammatory applications should consider multiple factors, including safety, biocompatibility, cost-effectiveness, specific use cases, and concentration requirements.

Limitations

It is crucial to highlight that both AgNPs and ZnONPs hold potential as anti-inflammatory agents, with their efficacy and suitability dependent on various factors. To fully understand the therapeutic potential and safety profiles of these nanoparticles in real-world anti-inflammatory applications, a lot of research is needed, including studies that take place inside living things. The encouraging results of recent studies show that AgNPs and green-synthesized ZnONPs could be useful tools in the fight against inflammation.

## Conclusions

In this in-depth study of green-synthesized nanoparticles, we looked at how to make herbal formulations and then make ZnONPs and AgNPs using extracts from *Ocimum tenuiflorum* (African basil) and *Ocimum gratissimum* (black tulsi). The herbal formulations, obtained by heating and filtration processes, served as key components in the green synthesis of nanoparticles. In the case of ZnONPs, the use of zinc nitrate and the subsequent centrifugation step yielded nanoparticles with potential anti-inflammatory properties. Similarly, the green synthesis of AgNPs using silver nitrate and herbal extracts showed that the process was eco-friendly and bioactively driven nanoparticle production.

The results show that both ZnONPs and AgNPs made from herbal extracts can reduce inflammation in a number of different tests. AgNPs displayed slightly higher inhibitory effects in some instances, particularly at lower concentrations, suggesting their efficacy as potential anti-inflammatory agents. The anti-inflammatory effects of these nanoparticles were not very different, though. This shows how important it is to look at the whole picture, including safety, biocompatibility, and cost-effectiveness for each application. The green synthesis approaches presented herein hold promise for diverse medical and therapeutic applications, underscoring the potential of herbal extracts in nanoparticle synthesis. These environmentally friendly approaches open doors to innovative and sustainable solutions in nanotechnology, with a focus on healthcare and pharmaceutical applications.
